# *Lactobacillus* supplementation for diarrhoea related to chemotherapy of colorectal cancer: a randomised study

**DOI:** 10.1038/sj.bjc.6603990

**Published:** 2007-09-25

**Authors:** P Österlund, T Ruotsalainen, R Korpela, M Saxelin, A Ollus, P Valta, M Kouri, I Elomaa, H Joensuu

**Affiliations:** 1 Department of Oncology, Helsinki University Central Hospital, PO Box 180, 00029 HUS Helsinki, Finland; 2 Institute of Biomedicine, University of Helsinki, PO Box 63, FI-00014 Finland; 3 Valio Ltd, R&D, PO Box 30, FI-00039 VALIO, Helsinki, Finland; 4 Department of Internal Medicine, Helsinki University Central Hospital, PO Box 340, FI-00029 Helsinki, Finland; 5 Department of Anaesthesiology, Helsinki University Central Hospital/Jorvi Hospital, Turuntie 150, FI-02740 Espoo, Finland

**Keywords:** chemotherapy, colorectal cancer, 5-fluorouracil, *Lactobacillus rhamnosus* GG, probiotic

## Abstract

5-Fluorouracil (5-FU)-based chemotherapy is frequently associated with diarrhoea. We compared two 5-FU-based regimens and the effect of *Lactobacillus* and fibre supplementation on treatment tolerability. Patients diagnosed with colorectal cancer (*n*=150) were randomly allocated to receive monthly 5-FU and leucovorin bolus injections (the Mayo regimen) or a bimonthly 5-FU bolus plus continuous infusion (the simplified de Gramont regimen) for 24 weeks as postoperative adjuvant therapy. On the basis of random allocation, the study participants did or did not receive *Lactobacillus rhamnosus* GG supplementation (1–2 × 10^10^ per day) and fibre (11 g guar gum per day) during chemotherapy. Patients who received *Lactobacillus* had less grade 3 or 4 diarrhoea (22 *vs* 37%, *P*=0.027), reported less abdominal discomfort, needed less hospital care and had fewer chemotherapy dose reductions due to bowel toxicity. No *Lactobacillus*-related toxicity was detected. Guar gum supplementation had no influence on chemotherapy tolerability. The simplified de Gramont regimen was associated with fewer grade 3 or 4 adverse effects than the Mayo regimen (45 *vs* 89%), and with less diarrhoea. We conclude that *Lactobacillus* GG supplementation is well tolerated and may reduce the frequency of severe diarrhoea and abdominal discomfort related to 5-FU-based chemotherapy.

Colorectal cancer is globally the third most common type of cancer and the fourth most common cause of cancer death (Parkin *et al*, 1999; Pisani *et al*, 1999). Curative surgery is feasible in three-quarters of the patients, but despite this, about one half of the patients subsequently develop incurable recurrent cancer (Galandiuk *et al*, 1992). Adjuvant chemotherapy or chemoradiation reduces recurrences and mortality in colorectal cancer (Van Cutsem *et al*, 2002). Regimens containing 5-fluorouracil (5-FU) and leucovorin (LV) have been considered as standard adjuvant chemotherapy regimens in colorectal cancer (O'Connell *et al*, 1998; Wolmark *et al*, 1999; Kerr, 2001), and addition of oxaliplatin to 5-FU and LV appears to further improve efficacy (Andre *et al*, 2004).

Diarrhoea is one of the most troublesome adverse effects related to cancer chemotherapy. 5-Fluorouracil-, capecitabine-, and irinotecan-based regimens that are commonly used in the treatment of colorectal cancer are frequently associated with diarrhoea. Excessive bowel motility may be reduced using drugs such as loperamide and somatostatin analogues, but these treatments may also be associated with adverse effects, and simple and safe measures to reduce drug-induced diarrhoea are thus needed. The mode of chemotherapy administration may also influence chemotherapy-related toxicity. Regimens where 5-FU is administered as protracted continuous infusions may result in a more favourable toxicity profile including the frequency and severity of diarrhoea as compared to the Mayo regimen, where 5-FU is given as boluses on 5 consecutive days 4-weekly (de Gramont *et al*, 1997a).

According to a meta-analysis of controlled trials performed on hospitalised children who have acute diarrhoea, co-administration of some microorganisms (probiotics) such as *Lactobacillus rhamnosus* GG with standard rehydration therapy reduces the duration of diarrhoea (Huang *et al*, 2002). Some placebo-controlled studies also suggest that probiotics are of benefit in the treatment of antibiotics-associated diarrhoea and in the prevention of nosocomial diarrhoea in infants (Szajewska *et al*, 2001; Cremonini *et al*, 2002). The putative mechanisms of *L. rhamnosus* GG action may include stimulation of the cell proliferation rate of bowel epithelial cells, enhanced secretion of protective mucins leading to reduced adherence of enteropathogenic bacteria to the bowel wall, inhibition of bacterial translocation into the tissues, and stimulation of local and systemic immune response to pathogens (Mattar *et al*, 2001; Banasaz *et al*, 2002; Khaled *et al*, 2003; Mack *et al*, 2003; Vaarala, 2003). Partially hydrolysed guar gum fibre may also reduce duration of diarrhoea (Homann *et al*, 1994; Alam *et al*, 2000) and prolong the colonic transit time (Meier *et al*, 1993). Thus, hypothetically, besides the mode of 5-FU administration, the frequency and severity of chemotherapy-associated gastrointestinal adverse events might be influenced by the diet and the bowel microbial flora.

Three studies have suggested that *Lactobacillus acidophilus*, *L. rhamnosus,* or a probiotic mixture may prevent radiotherapy-induced diarrhoea (Salminen *et al*, 1988; Urbancsek *et al*, 2001; Delia *et al*, 2002), but to our knowledge no controlled study has evaluated probiotics or fibre in the prevention of chemotherapy-associated diarrhoea. In the present study, we assessed the efficacy of *L. rhamnosus* GG and guar gum supplementation in reducing 5-FU-based chemotherapy toxicity. We also compared the tolerability and the frequency of diarrhoea related to the Mayo regimen to that of the simplified de Gramont regimen, which uses a bolus plus continuous 5-FU infusion.

## METHODS

### Study design and accrual

The primary end point of this open-label, prospective, randomised, phase III, single institution, 2 × 3 factorial design study was the frequency of severe diarrhoea. The study participants had either Dukes' B or C colorectal cancer (*n*=126) or metastatic colorectal cancer that had been rendered free from all overt metastases by surgery (Dukes' D, *n*=24). All patients received adjuvant chemotherapy following surgery. Chemotherapy consisted either of the Mayo regimen or the simplified de Gramont regimen, and was administered based on random allocation. In addition, study participants diagnosed with rectal cancer received locoregional radiotherapy whenever the caudal tumour margin was below the distal peritoneal fold.

One hundred and fifty-four subjects were assessed for the study between November 1997 and August 2001. Of these, one was ineligible due to age and three others preferred not to participate leaving a total of 150 eligible patients who consented to participate in the study. An Institutional Review Board at Helsinki University Central Hospital approved the study protocol prior to initiation of the study. A written informed consent was required from the participants prior to study entry.

### Treatment assignment

Allocation to the study treatments was performed using a computerised minimisation technique (Pocock and Simon, 1975; Freedman and White, 1976) and one out of six chances. The patients were randomly allocated at a 1 : 1 ratio to receive either the simplified de Gramont regimen or the Mayo regimen as adjuvant chemotherapy. The participants were also randomly assigned to receive or not to receive at a 2 : 1 ratio *L. rhamnosus* GG and at a 1 : 2 ratio fibre-containing nutritional support (guar gum). The allocation group was concealed until interventions had been assigned. The patients were stratified by gender, tumour site (colon or rectum), and the Dukes' stage at randomisation.

### Participant eligibility

Subjects were eligible for inclusion provided that age at randomisation was 18 or higher and 75 or lower, histologically confirmed colorectal cancer had been removed at surgery, no metastases were found in staging examinations that included ultrasound or computed tomography (CT) of the abdomen and chest X ray or CT of the thorax, and the World Health Organization (WHO) performance status was two or less. The study participants were also required to have adequate bone marrow, kidney, and liver functions for chemotherapy. Exclusion criteria included other invasive cancer beside colorectal cancer in history except for carcinoma *in situ* of the cervix or nonmelanoma skin cancer; metabolic, neurological, or psychiatric disease that was incompatible with chemotherapy; a serious thromboembolic event under active treatment; and pregnancy, lactation, or absence of adequate contraception in potentially fertile patients.

### Adjuvant systemic chemotherapy and radiation therapy

The Mayo regimen consisted of a short intravenous infusion of LV 20 mg m^−2^ (or 10 mg m^−2^ levoleucovorin) and 5-FU 370–425 mg m^−2^ administered as an intravenous bolus over 3–5 min on days 1–5 of the cycle, which was repeated at 4-week intervals for six times. The overall duration of chemotherapy was thus 24 weeks.

The simplified de Gramont regimen consisted of a 2-h infusion of LV 400 mg m^−2^ (or 200 mg m^−2^ levoleucovorin) followed by 5-FU 400 mg m^−2^ administered as an intravenous bolus and 48-h infusion of 3.0–3.6 g m^−2^ 5-FU; this cycle was repeated every 14 days for 12 times. The overall duration of chemotherapy was thus 24 weeks (de Gramont *et al*, 1997b).

Pelvic radiation therapy for rectal cancer was given from three or four portals using high-energy photons obtained from a linear accelerator (*n*=39). Radiotherapy, based on CT planning, was administered to a total cumulative dose of 50.4 Gy in 1.8 Gy daily fractions over 5.5 weeks except for patients who underwent abdominoperineal resection, when the dose was limited to 45 Gy to decrease the likelihood of small bowel radiation injury (*n*=8). Leucovorin was omitted during pelvic radiotherapy (cycles three and four) in rectal cancer patients assigned to receive the Mayo regimen, and bolus 5-FU, 500 mg m^−2^, was given only for 3 days during these cycles. Similarly, rectal cancer patients assigned to receive the simplified de Gramont regimen did not receive LV during pelvic radiotherapy (cycles five to eight) and were treated with continuous 5-FU infusion alone, 225 mg m^−2^ per day (O'Connell *et al*, 1994). Seven rectal cancer patients were treated to a cumulative target dose of 25 Gy given as five equal fractions over 5 consecutive days preoperatively (Anonymous, 1996; Kapiteijn *et al*, 2001); no concomitant 5-FU was given to these patients during radiation, and their postoperative systemic chemotherapy was given as for colon cancer patients.

### Dietary supplementation

*Lactobacillus rhamnosus* GG (ATCC 53103, Gefilus®, Valio Ltd, Helsinki, Finland) was administered orally as gelatine capsules twice daily at a dose of 1–2 × 10^10^ per day during the 24 weeks of adjuvant cancer chemotherapy. *Lactobacillus* capsules were swallowed as such, or the capsule content was dissolved in cold milk or juice. Guar gum containing nutritional supplement (500 ml, Novasource GI control®, Novartis Nutrition, Basel, Switzerland (contains 11 g guar gum and 550 kcal or 2300 kJ), was administered daily, on cycle days 7–14, for 8 days per month. All patients received dietary counselling.

### Concomitant medications

No other dietary supplements were allowed during the study. Treatment compliance was monitored using nutrition diaries. Prophylactic antibiotics or leukocyte growth factors were not routinely prescribed during chemotherapy, but the patients were allowed to use any medication deemed necessary for appropriate care of concomitant diseases. Metoclopramide and 5-HT3 inhibitors were used for nausea/vomiting, loperamide for diarrhoea, dexpanthenol lozenges 100–200 mg t.i.d. for stomatitis, and pyridoxine 50 mg t.i.d. for hand–foot syndrome at the discretion of the treating physician.

### Monitoring of treatment-related adverse effects

Patients were scheduled to be evaluated within 21 days prior to study treatment initiation, 4 weekly during chemo- and radiotherapy, and at protocol-determined intervals (ranging from 2 to 6 months) posttreatment. At each visit, medical history was taken and physical examination was performed, which included the WHO performance status and weight, and the blood cell counts and serum chemistry were analysed. Blood cell counts were monitored at 10 to 14-day intervals. Treatment-related adverse effects were evaluated at every cycle using a diary kept by the patients and by a physician (PÖ or TR). Adverse events were assessed and graded according to the Common Toxicity Criteria of the National Cancer Institute of Canada scale version 2.

### Number of patients, power assumptions, and statistical methods

No reliable estimation of *L. rhamnosus* GG efficacy in reduction of chemotherapy-associated diarrhoea could be made prior to the study due to lack of relevant data. The frequency of grades 3 and 4 diarrhoea was the primary variable. Assuming a 50% reduction in the frequency of grade 3 or 4 diarrhoea from 40–50% to 20–25%, using 2 : 1 allocation between the study arms, an 80% power, and a 0.05 significance level, approximately 150 patients need to be accrued to the study. On the basis of prior data (de Gramont *et al*, 1997a), patients who receive continuous 5-FU might have less adverse effects than those treated with bolus 5-FU. A two-group *χ*^2^ test at the 0.05 significance level (two-sided) has an 80% power to detect a difference in the frequency of diarrhoea between the proportions of 0.60 or 0.40 in the bolus regimen group and proportions of 0.35 or 0.20 in the continuous 5-FU administration group, when the respective sample sizes are 62 and 82, also suggesting that accrual of approximately 150 patients allows detection of approximately 20% difference in the rate of diarrhoea between the groups. No interactions between chemotherapy and nutritional supplements were assumed.

The study was analysed according to the intention-to-treat principle, and outcome was analysed as defined in the study protocol. The statistical analyses were performed with a StatView computer program (SAS institute, Abacus concepts incorporation, Berkeley, CA, USA). The effects of bolus 5-FU *vs* continuous 5-FU regimens, chemoradiation *vs* chemotherapy only, *L. rhamnosus* GG *vs* no dietary supplements, and guar gum *vs* no guar gum were compared using univariate and multivariate logistic regression models. The results are given as odds ratios with 95% confidence intervals. Mann–Whitney *U*-test was used to compare the treatment groups with respect to quantitative response variables. Frequency tables were analysed using the *χ*^2^ test.

## RESULTS

### Patient characteristics and compliance

The treatment arms were balanced with gender, the WHO performance status, primary tumour site, Dukes' stage, and radiation therapy given (Table 1). The median age at randomisation was 60 (range: 31–75); 51% of the participants were men. Sixteen (11%) subjects did not complete the scheduled 6 months of adjuvant chemotherapy due to either adverse events (*n*=7, six of whom received bolus 5-FU), cancer recurrence (*n*=5), or a concomitant disease (*n*=4). Two patients (both in the continuous 5-FU group) who did not receive any of the study treatments due to postoperative complications were not included in safety or efficacy analyses leaving 148 patients for these analyses. None of the patients were lost to follow-up.

### Chemotherapy dose intensity and tolerability

The scheduled 5-FU dose intensity, calculated as the percentage of the scheduled dose as milligrams of 5-FU given per square metre per week of the scheduled cumulative dose, was maintained better among the patients who received the simplified de Gramont regimen than among those treated with the Mayo regimen (median 93 *vs* 78%, respectively; *P*<0.0001).

The simplified de Gramont regimen was tolerated better than the Mayo regimen (Table 2). Any grade 3 or 4 adverse effect was present in 87% (65 out of 75) of the patients treated with the Mayo regimen as compared with only 45% (33 out of 73) of those treated with the simplified de Gramont regimen, when the vascular access device (VAD)-related toxicity was included in this analysis (*P*<0.0001, Table 2). Vascular access device-related complications occurred in seven (10%) patients who received continuous 5-FU infusions; three were classified as serious. The Mayo regimen was more frequently associated with stomatitis, diarrhoea, and neutropenia than the simplified de Gramont regimen, whereas mild-to-moderate hand–foot syndrome was more common among the patients who received the simplified de Gramont regimen (*P*<0.0001). There were no treatment-related deaths.

### *Lactobacillus* supplementation

**Figure 1:**
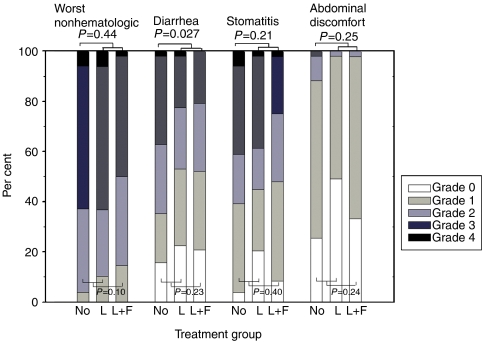
Effect of oral *Lactobacillus rhamnosus GG* (L) and *Lactobacillus rhamnosus GG* plus fibre (guar gum, L+F) supplementation on adverse events recorded during 5-FU-based chemotherapy.

Forty-nine (51%) of the 97 patients randomly allocated to receive oral *Lactobacillus* supplementation and 26 (51%) of the 51 patients who were allocated to the control group were treated with the Mayo regimen (*P*=0.96). Compliance to *Lactobacillus* supplementation was excellent, and all patients consumed their scheduled doses. The frequency of grade 3–4 diarrhoea was lower among patients who received *Lactobacillus* supplements than in the rest of the patients (22 *vs* 37%; *P*=0.027, Figure 1). Seventy-six (78%) patients in the *Lactobacillus* supplementation group and 43 (84%) in the control group reported diarrhoea of any grade during the study (tested grade 0 *vs* >0, *P*=0.39). Abdominal discomfort resulting from flatulence, borborygmia, or abdominal distension was less in patients who received *Lactobacillus* supplements (grade 2 or 3 in 2 *vs* 12%, *P*=0.025), and any grade of abdominal discomfort was present in 57 (59%) of the patients who received *Lactobacillus* versus in 38 (75%) of those who did not (*P*=0.058). However, *Lactobacillus* supplementation had no significant effect on the overall toxicity of treatment, or the frequency of stomatitis or neutropenia (Table 2).

None of the patients had *Lactobacillus* GG growth in blood bacterial cultures. Nine (10%) patients allocated to receive *Lactobacillus* had neutropenic infection as compared to two (4%) of those who did not receive it (*P*=0.24). Eight patients (8%) in the *Lactobacillus* group required hospital care for bowel toxicity, as compared to 11 (22%) in the comparator group (*P*=0.021). Twenty (21%) of the patients who received *Lactobacillus* supplementation had chemotherapy-dose reductions due to bowel toxicity as compared to 24 (47%) among those who did not receive *Lactobacillus* (*P*=0.0008). Only one patient discontinued chemotherapy primarily due to bowel toxicity (this patient was allocated not to receive *Lactobacillus* supplementation).

### Fibre supplementation

Nine (18%) patients discontinued fibre supplementation due to a taste aversion (these patients were included in the analysis according to the intention-to-treat principle). Addition of fibre did not influence the overall gastrointestinal toxicity (*P*=0.13). Guar gum supplementation did not reduce the frequency of severe diarrhoea as compared to patients who did not receive fibre (25 *vs* 30%, respectively; *P*=0.24). There was no difference between the allocation groups in the proportions of patients who had abdominal discomfort resulting from flatulence, borborygmia, or abdominal distension (grade 2 or 3 in 2 *vs* 7%, *P*=0.24), and the rates of chemotherapy-dose reductions due to bowel toxicity were also similar between the groups (*P*=0.20).

## DISCUSSION

Chemotherapy-related diarrhoea is a common adverse effect in the treatment of colorectal cancer, since for example 5-FU, capecitabine, and irinotecan administration is frequently associated with diarrhoea. Severe diarrhoea may lead to nutritional and metabolic imbalances, and severe neutropenia associated with diarrhoea may be life threatening. The present findings indicate that the frequency of grade 3 or 4 diarrhoea may be reduced with the use of *Lactobacillus* supplementation. The latter finding is of interest, since *Lactobacillus* supplementation appears to have few or no adverse effects, *Lactobacillus* capsules are simple to administer, and they are associated with low costs. Patients who received *Lactobacillus* during chemotherapy reported less abdominal discomfort than those who did not receive it, and these subjects had also fewer chemotherapy-dose reductions, which might have an impact on chemotherapy efficacy. As many other bacteria, lactobacilli may occasionally cause septicaemia in severely immunocompromised patients (Salminen *et al*, 2004), but *L. rhamnosus* was identified in none of the blood cultures during the study. There was no difference between the allocation groups in the frequency of neutropenic fever.

Somewhat unexpectedly, nutritional supplements have not been evaluated in the prevention and treatment of chemotherapy-related gastrointestinal adverse effects in controlled studies. In one study, dosing of *Lactobacillus plantarum* during 5-FU administration improved food intake and helped to maintain the body weight in rats, but it did not prevent diarrhoea (Von Bultzingslowen *et al*, 2003). Instead, probiotics have been studied in a variety of bowel diseases other than cancer in humans. *Lactobacillus rhamnosus* GG has been found to alleviate diarrhoea caused by a viral infection or *Clostridium difficile*, and to be useful in the prevention of traveller's diarrhoea or diarrhoea related to administration of antibiotics (Ouwehand *et al*, 2002; Vaarala, 2003). Probiotics may also reduce radiation therapy-related diarrhoea (Salminen *et al*, 1988; Urbancsek *et al*, 2001; Delia *et al*, 2002). The mode of action is not fully understood, but probiotics are involved in some cytoprotective processes, such as induction of heat-shock protein expression in intestinal epithelial cells (Tao *et al*, 2006), and prevention of cytokine-induced epithelial cell damage (Yan *et al*, 2007).

The combination of hydrolysed guar gum fibre and lactobacilli has been suggested to be effective for diarrhoea (Meier *et al*, 2003). One-third of the present patients received this combination, but we detected no further reduction in gastrointestinal adverse effects among patients who received the combination. The optimal dose and schedule to administer fibre are not known. We administered 11 g hydrolysed guar gum fibre daily for 8 days per month, but this dose may have been too low or the intervention duration too short. We chose not to administer guar gum concomitantly with chemotherapy to avoid nausea, because some individuals find its taste aversive.

The simplified de Gramont regimen that includes 5-FU given both as a bolus and as a 48-h continuous infusion was found to be better tolerated than the Mayo regimen, where 5-FU is given as boluses only. Earlier comparisons involving continuous and protracted 5-FU regimens are in line with present findings suggesting that the more protracted regimens are generally associated with less adverse effects (de Gramont *et al*, 1997a; Andre *et al*, 2003; Kohne *et al*, 2003; Saini *et al*, 2003). Efficacy comparisons between the de Gramont regimen or its modifications and the Mayo regimen suggest that the former are no less effective (de Gramont *et al*, 1997a; Andre *et al*, 2003).

Grade 3 or 4 adverse effects were frequently reported in the present series; 45% of the patients treated with the simplified de Gramont regimen and 87% of those treated with the Mayo regimen had at least one grade 3 or 4 adverse effect. These figures are higher (Andre *et al*, 2003) or approximately similar in frequency (Labianca *et al*, 1995) as those reported from studies where similar types of chemotherapy have been administered in the adjuvant setting. Use of radiation therapy in rectal cancer, and the patients keeping a diary may have increased the number of reported events in the present series. We administered 5-FU as true boluses with an approximate injection time of 3 min, which may be associated with more adverse events than short (about 15 min) infusions (Andre *et al*, 2001). We also included VAD-related adverse effects, though they were infrequent in comparison to some other series (Carde *et al*, 1989; Gleeson *et al*, 1993; Kock *et al*, 1998).

The chemotherapy regimens investigated did not contain irinotecan, capecitabine, or oxaliplatin that are now commonly used to treat colorectal cancer, which is a limitation of the study. The study was not placebo-controlled nor blinded to administration of the dietary supplements, which may or may not have influenced assessment of adverse effects. To remedy these potential shortcomings, we have initiated a prospective, randomised, multicentre, double blind, placebo-controlled study with a cross-over design where we investigate the effects of *Lactobacillus* supplementation in conjunction with chemotherapy that contains capecitabine, oxaliplatin, irinotecan, and bevacizumab.

We conclude that daily oral administration of *L. rhamnosus* GG may reduce the frequency of severe 5-FU-based chemotherapy-related diarrhoea, whereas fibre supplementation may be of little benefit. *Lactobacillus* supplementation may be a practical and well-tolerated means to reduce the severity of 5-FU-based chemotherapy-induced diarrhoea, and deserves to be evaluated further.

## COMPETING INTERESTS

TR, AO, PV, MK, IE, and HJ declare no conflict of interest. PÖ has received an honorarium from Baxter for teaching. MS is an employee at Valio Research center, Valio Ltd., Finland. RK is an employee at Valio Research center and University of Helsinki, Institute of Biomedical Sciences. This is an investigator-initiated and conducted study. *Lactobacillus rhamnosus* GG capsules were provided free-of-charge for the study by Valio. Novasource GI control and infusing devices were purchased from Novartis Finland and Baxter Finland at a discounted price for the study. The investigators collected all study data, had access to all data, and wrote the manuscript.

## Figures and Tables

**Table 1:** Patient characteristics

**Characteristic**	**Entire series *N*=150 *n* (%)**	**Bolus 5-FU/LV *N*=75 *n* (%)**	**Continuous 5-FU/LV *N*=75 *n* (%)**	**Lactobacillus not given *N*=52 *n* (%)**	**Lactobacillus given *N*=98 *n* (%)**
Median age (range)	60 (31–75)	61 (35–75)	59 (31–73)	57 (31–75)	61 (35–74)
					
*Gender*					
Male	76 (51)	39 (52)	37 (49)	25 (48)	51 (52)
Female	74 (49)	36 (48)	38 (51)	27 (52)	47 (48)
					
*Site*					
Colon	90 (60)	44 (59)	46 (61)	31 (60)	59 (60)
Rectum	60 (40)	31 (41)	29 (39)	21 (40)	39 (40)
					
*Dukes' stage*					
B	40 (27)	20 (27)	20 (27)	13 (25)	27 (28)
C	86 (57)	42 (56)	44 (59)	31 (60)	55 (56)
D^a^	24 (16)	13 (17)	11 (15)	8 (15)	16 (16)
					
*Rectal radiotherapy*					
Not given	96 (64)	48 (64)	46 (61)	33 (63)	63 (64)
Given	54 (36)	27 (36)	27 (39)	19 (37)	35 (36)

Abbreviations: 5-FU=5-fluorouracil; LV=leucovorin.

a Patients were rendered free from all macroscopic cancer by surgery.

**Table 2:** Treatment-related adverse effects

**Adverse effect**	**Bolus 5-FU/LV *N*=75 *n* (%)**	**Continuous 5-FU/LV *N*=73 *n* (%)**	**Odds ratio (95% CI)**	**No Lactobacillus *N*=51 *n* (%)**	**Lactobacillus given *N*=97 *n* (%)**	**Odds ratio (95% CI)**
*Any*						
Grade 0–2	10 (13)	40 (55)	0.14 (0.06–0.31)	16 (31)	34 (35)	0.77 (0.35–1.72)
Grade 3–4	65 (87)	33 (45)	*P*<0.0001	35 (69)	63 (65)	*P*=0.53
						
*Stomatitis*						
Grade 0–2	32 (43)	64 (88)	0.09 (0.04–0.22)	30 (59)	66 (68)	0.59 (0.26–1.35)
Grade 3–4	43 (57)	9 (12)	*P*<0.0001	21 (41)	31 (32)	*P*=0.21
						
*Diarrhoea*						
Grade 0–2	42 (56)	66 (90)	0.11 (0.04–0.29)	32 (63)	76 (78)	0.38 (0.16–0.89)
Grade 3–4	33 (44)	7 (10)	*P*<0.0001	19 (37)	21 (22)	*P*=0.027
						
*Neutropenia*						
Grade 0–2	53 (71)	63 (86)	0.38 (0.16–0.90)	43 (84)	73 (75)	2.00 (0.74–4.89)
Grade 3–4	22 (29)	10 (14)	*P*=0.029	8 (16)	24 (25)	*P*=0.19
						
*Neutropenic infection*						
No	67 (89)	70 (96)	0.34 (0.08–1.4)	49 (96)	88 (90)	2.62 (0.53–13)
Yes	8 (11)	3 (4)	*P*=0.13	2 (4)	9 (10)	*P*=0.24
						
*Hand–foot syndrome*						
Grade 0–2	75 (100)	70 (96)	−(0.0–	50 (98)	95 (98)	−(0.0–
Grade 3	0 (0)	3 (4)	*P*=0.98	1 (2)	2 (2)	*P*=0.97

Abbreviations: CI=confidence interval; 5-FU=5-fluorouracil; LV=leucovorin.
